# Activation of Human Complement System by Dextran-Coated Iron Oxide Nanoparticles Is Not Affected by Dextran/Fe Ratio, Hydroxyl Modifications, and Crosslinking

**DOI:** 10.3389/fimmu.2016.00418

**Published:** 2016-10-10

**Authors:** Guankui Wang, Fangfang Chen, Nirmal K. Banda, V. Michael Holers, LinPing Wu, S. Moein Moghimi, Dmitri Simberg

**Affiliations:** ^1^Department of Pharmaceutical Sciences, Skaggs School of Pharmacy and Pharmaceutical Sciences, University of Colorado Anschutz Medical Campus, Aurora, CO, USA; ^2^Department of Gastrointestinal Surgery, China-Japan Union Hospital, Jilin University, Changchun, China; ^3^Division of Rheumatology, School of Medicine, University of Colorado Denver, Aurora, CO, USA; ^4^Nanomedicine Laboratory, Department of Pharmacy, Centre for Pharmaceutical Nanotechnology and Nanotoxicology, University of Copenhagen, Copenhagen, Denmark; ^5^School of Medicine, Pharmacy and Health, Durham University, Durham, UK

**Keywords:** iron oxide nanoparticles, complement C3, complement system proteins, properdin, dextran, lectin pathway, alternative pathway of complement

## Abstract

While having tremendous potential as therapeutic and imaging tools, the clinical use of engineered nanoparticles has been associated with serious safety concerns. Activation of the complement cascade and the release of proinflammatory factors C3a and C5a may contribute to infusion-related reactions, whereas opsonization with C3 fragments promotes rapid recognition and clearance of nanomaterials by mononuclear phagocytes. We used dextran-coated superparamagnetic iron oxide nanoparticles (SPIO), which are potent activators of the complement system, to study the role of nanoparticle surface chemistry in inciting complement in human serum. Using complement inhibitors and measuring levels of fluid phase markers (sC5b-9, C5a, and Bb), we found that the majority of human complement activation by SPIO is through the alternative pathways (AP). SPIO prepared with high dextran/iron ratio showed some complement activation *via* calcium-sensitive pathways, but the AP was responsible for the bulk of complement activation and amplification. Activation *via* the AP required properdin, the positive regulator of the alternative C3bBb convertase. Modification of sugar alcohols of dextran with alkylating, acylating, or crosslinking agents did not overcome complement activation and C3 opsonization. These data demonstrate that human complement activation is independent of dextran modification of SPIO and suggest a crucial role of the AP in immune recognition of nano-assemblies in human serum.

## Introduction

Complement system is a critical component of the innate immunity that comprises ~5% of globulins and is responsible for eliminating and destroying pathogens (1). Complement activation *via* classical, lectin, and alternative pathways (AP) converge to form highly reactive thioester C3b that covalently binds to hydroxyls and amines on foreign surfaces (2, 3) resulting in the formation of membrane pore complex C5b-9 and extremely potent anaphylatoxins C3a and C5a (4). Opsonization by C3b and its cleaved products (e.g., iC3b, C3d) triggers immune recognition by neutrophils, eosinophils, lymphocytes, monocytes, red blood cells, and macrophages (5, 6). Complement activation is also believed to contribute toward infusion-related reactions with clinically approved nanopharmaceuticals, such as Doxil (liposomal doxorubicin), Taxol (Cremophor-paclitaxel), and Sandimmune (Cremophor-cyclosporine A).

Despite the fact that numerous reports demonstrated complement activation by nanoparticles, liposomes, and micelles (7–22), the pathways of complement activation as function of surface properties are still poorly understood. One of the examples is superparamagnetic iron oxide (SPIO) nanoparticle, which is widely used not only as a contrast agent in magnetic resonance imaging (MRI) but also in the development of theranostic nanomedicines and experimental hyperthermia treatments (23). Previously, others and we described the preparation of high contrast SPIO nanoworms (SPIO NWs) (24–28) that consist of multiple Fe_3_O_4_ crystals embedded in 20 kDa linear dextran. We further reported that dextran-coated SPIO NWs activate complement in mouse serum *via* the lectin pathway, but in human serum complement activation is *via* lectin and APs (24–28). Furthermore, others (3, 29–31) have pointed out that dextran-coated particles consume complement, where the projected surface polymer in brush conformation is less efficient in complement consumption than a side-on conformation. In addition, it has been reported that crosslinked dextran (Sephadex) enhances complement activation, and substitution of alcohol groups can partially prevent this effect (32–34). Despite these advances, the effect of carbohydrate modifications of dextran-coated SPIO on the efficiency of complement activation has not been investigated. This knowledge is not only very critical for SPIO nanoparticles, which are clinically useful nanomaterials, but also for surface engineering of other carbohydrate-coated materials. Indeed, several iron oxide-based clinical contrast agents, such as Feridex and Combidex, have induced adverse reactions in a large number of patients, presumably as a result of complement activation.

Here, we prepared SPIO using different dextran/Fe ratios and studied the pathway of complement activation by measuring generation of fluid phase markers. Our results point to the critical role of the AP in complement activation by SPIO regardless of the dextran/Fe ratio and the nanoparticle size. We then used SPIO NWs prepared with low dextran/Fe ratio to further understand the effect of modification of sugar hydroxyls with alkylating and crosslinking agents on C3 opsonization. The results suggest that modifications of dextran coat are not an effective strategy to mitigate AP activation by these nanoparticles in humans.

## Results

### Alternative Pathway Is the Main Activation Pathway by SPIO Regardless of Dextran/Fe Ratio and Particle Size

**Figure 1:**
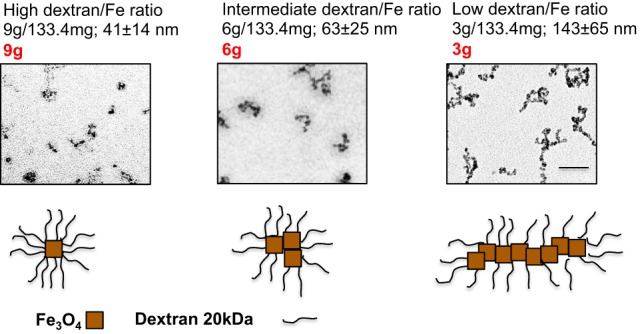
**Preparation of SPIO NWs with different dextran/Fe ratios: the particles were prepared with different ratios of dextran to Fe salt**. The size of the SPIO aggregates increases as the ratio used in the precipitation reaction decreases, from single crystal (9 g) to smaller aggregates of several crystals (6 g) to polycrystalline (5–20 crystals) nanoworms (3 g).

We synthesized SPIO nanoparticles by mixing 20 kDa dextran with FeCl_2_ and FeCl_3_ and precipitating nanoparticles with ammonia (35). For the precipitation reaction, we used different dextran/Fe ratios (low ratio: 3.0 g/133.4 mg; intermediate ratio: 6.0 g/133.4 mg; high ratio: 9.0 g/133.4 mg). Hydrodynamic size measurements (Figure 1) showed that particles prepared at intermediate and high dextran/Fe ratios (6 and 9 g dextran, respectively) were much smaller than particles prepared at low dextran/Fe ratio (3 g dextran), apparently due to a more efficient coating of individual crystals with dextran and prevention of intercrystal aggregation. Transmission electron microscopy (TEM) images (Figure 1) showed that SPIO particles prepared with higher dextran/Fe ratio were rounded with few crystalline Fe_3_O_4_ cores (Figure 1, bottom schematic), whereas SPIO prepared with low dextran/Fe ratio (3 g) were predominantly polycrystalline worm-like structures (we term them SPIO nanoworms or SPIO NWs).

**Figure 2:**
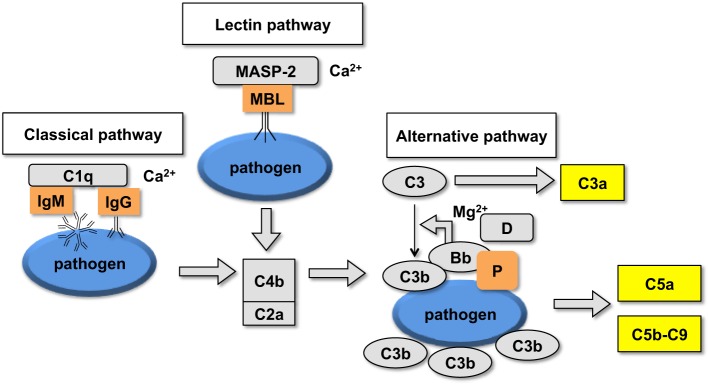
**Schematic representation of the upstream part of the complement cascade: assembly of different pathways on the foreign surface leads to the formation of complement convertases and generation of C3b and fluid phase markers**.

**Figure 3:**
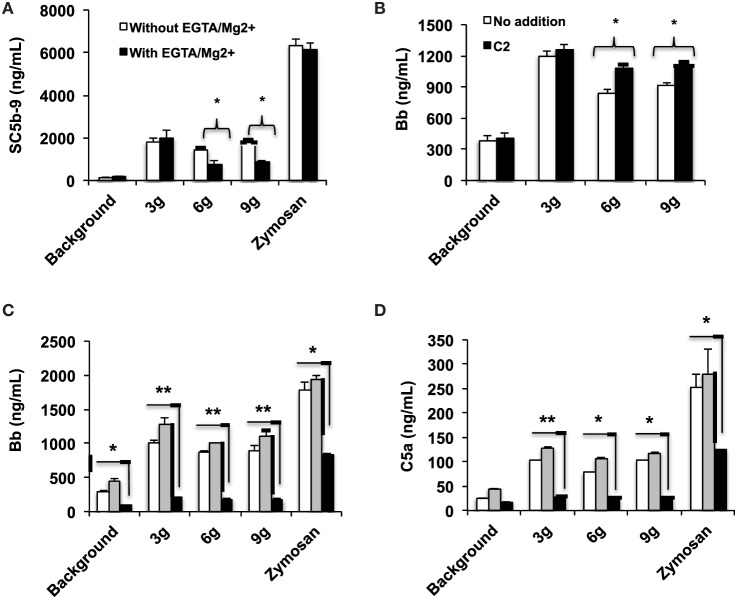
**Mechanisms of complement activation studied with fluid phase markers: formulations of SPIO NWs are described in Figure 1 (dextran/Fe ratios abbreviated as 3, 6, and 9 g) were incubated in human sera at 0.2 mg Fe/mL as described in Section “Materials and Methods”**. Zymosan (positive control) was at 0.2 mg/mL; **(A)** generation of soluble terminal membrane attack complex sC5b-9 in a healthy human serum; **(B)** generation of Bb as a marker of the AP activation in C2-depleted serum [the same serum source as in **(A)**, where C2 was depleted immunochemically] and after addition of recombinant C2 (650 μg/mL) to C2-depleted serum; **(C,D)** effect of properdin antibody on AP activation and C5a generation, respectively. White bars: no inhibitor, gray bars: control isotype matched antibody, black bars: anti-P antibody. Non-parametric two sided *t*-test, *n* = 3 (two different human sera were used, and the results of a typical experiment are presented); *p < 0.05; **p < 0.01. None of the SPIO NWs generated sC5b and Bb in the presence of 10 mM EDTA (not shown).

The human complement (Figure 2) is triggered by foreign surfaces *via* the formation of activated C3(H_2_O) (AP turnover) or *via* calcium-sensitive pathways (classical or lectin). This activation leads to the initially deposited C3b that associates with factor B to form the AP convertase C3bBb, which cleaves additional C3 molecules. In order to study the pathway of complement activation, nanoparticles were added to human serum at Fe concentration of 0.2 mg/mL. Measurement of the soluble terminal complex marker sC5b-9 showed that all formulations triggered complement to the same extent, regardless of the size and dextran/Fe ratio (Figure 3A). Addition of calcium chelator 10 mM EGTA/2.5 mM Mg^2+^ [to inhibit operation of calcium-sensitive pathways (36)] dramatically decreased (by 40%) complement activation by NWs prepared with high and intermediate dextran/Fe ratios, but not by NWs prepared with low dextran/Fe ratio (Figure 3A). These data suggest that at higher dextran/Fe ratios, both calcium-sensitive and the APs contribute to complement activation, whereas at low dextran/Fe ratio, the activation proceeds exclusively *via* the AP. In order to investigate the contribution of calcium-sensitive pathways in formation of the AP convertase (Figure 2), we measured generation of Bb in sera deficient in C2, which is the critical factor for calcium-sensitive pathways. According to Figure 3B, SPIO NWs showed no decrease in Bb in the absence of C2, whereas 6 and 9 g SPIO showed a 25% decrease in Bb in the absence of C2. Bb levels were restored to normal levels when C2, at a physiological concentration (650 μg/mL), was added to the depleted serum. Collectively, these experiments confirm that calcium-sensitive pathways contribute to the complement activation by NWs with more polysaccharide content. In order to understand to what extent complement activation can proceed in the absence of the AP, we tested Bb and C5a formation in presence of anti-properdin (P) antibodies. The AP convertase is stabilized by P, being present in blood at ~20 μg/mL. For all formulations regardless of the dextran/Fe ratio, anti-P blocking antibody [a potent blocker of the AP (37)] inhibited AP convertase generation by over 80% (Figure 3C) and prevented C5a release by over 70% (Figure 3D), Collectively, these data suggest that despite contribution of the calcium-sensitive pathways to the initiation of complement and formation of the AP convertase by particles prepared at high dextran/Fe ratio, the AP still plays a predominant role on the propagation of complement and generation of fluid phase markers for all tested formulations.

### Surface Modifications of SPIO NWs Do Not Decrease Complement Activation and C3 Opsonization

**Figure 4:**
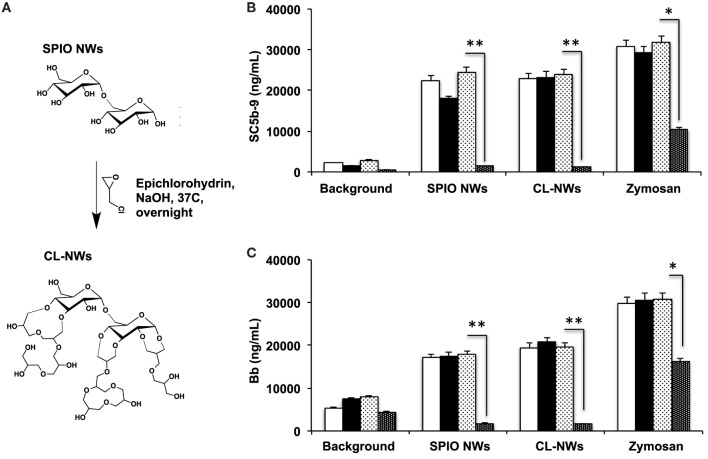
**Effect of crosslinking of SPIO NWs on the complement activation as measured with fluid phase markers**. **(A)** Scheme of crosslinking as described previously (35) leads to the formation of 3D crosslinked hydrogel on the particle surface; **(B,C)** generation of membrane attack complex and Bb, respectively, in human serum. Particle concentration was 0.4 mg/mL, zymosan concentration was 0.2 mg/mL. White bars: no inhibitor; black bars: EGTA/Mg^2+^; light dotted bars: control isotype matched antibody; dark dotted bars: anti-P antibody. The crosslinking of dextran did not block complement activation and did not change the pathway of activation (AP). Non-parametric two-sided *t*-test, *n* = 3; *p < 0.05; **p < 0.01 (two different human sera were used, and the results of a typical experiment are presented).

Because 3 g SPIO NWs show only a single pathway of complement activation, as opposed to 6 and 9 g SPIO that exhibit also calcium-dependent activation, in the subsequent studies, we studied the effect of surface modifications of SPIO NWs on complement activation. SPIO NWs are highly efficient MRI contrast agents (35), and their opsonization by C3 leads to immune cell uptake (38), therefore, strategies to block complement activation would have a great value in the translation of these particles. Previously, we reported that crosslinking dextran coat of SPIO NWs with epichlorohydrin (resulting in CL-NWs, Figure 4A) blocked lectin pathway activation and C3 opsonization in mouse serum, but this procedure did not block C3 opsonization in sera from human subjects (35). In order to determine the pathway responsible for complement activation of CL-NWs in human serum, we measured the fluid markers sC5b-9 and Bb (Figures 4B,C). SPIO NWs and CL-NWs caused comparable AP activation that was not inhibited by 10 mM EGTA/2.5 mM Mg^2+^. At the same time, anti-P antibody, but not control antibody, blocked over 90% of sC5b-9 and Bb release for both SPIO NWs and CL-NWs. These results confirm that complement activation by CL-NWs proceeds almost exclusively *via* the AP.

**Figure 5:**
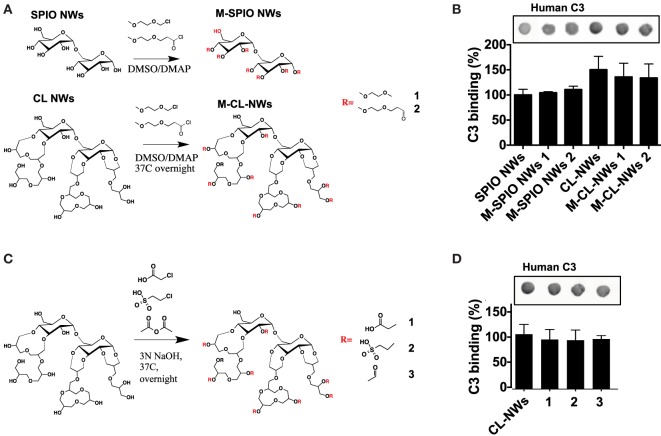
**Modifications of hydroxyl groups of SPIO NWs and CL-NWs do not decrease complement C3 opsonization**. **(A)** Alkylation and acylation of hydroxyls on SPIO NWs and CL-NWs; **(B)** modifications in **(A)** did not decrease or even increased C3 opsonization; **(C)** modification of CL-NW hydroxyls with acetyl, carboxymethyl, and sulfoethyl groups; **(D)** modifications in **(C)** did not significantly decrease C3 opsonization in human serum. Three different human sera were used in triplicates, and the results of a typical experiment are presented.

The AP is triggered by the initial deposition of C3b on a foreign surface *via* highly reactive thioester group that covalently attaches to amines and hydroxyls (39). Both dextran and crosslinked dextran contain hydroxyls available for nucleophilic attack of the thioester bond and subsequent deposition of C3b (Figure 4A). Previous report suggested that substituting hydroxyl groups could reduce complement consumption by Sephadex (crosslinked dextran beads) (32). In order to test the hypothesis whether substitution of dextran hydroxyls by alkylating and acylating agents could block complement activation, we modified hydroxyl groups of SPIO NWs and CL-NWs with an esterifying agent 2-(2-methoxyethoxy)acetyl chloride or etherifying agent 2-methoxyethoxymethyl chloride (Figure 5A) and measured C3 opsonization in human serum. According to Figure 5B, while there was a significant deposition of C3 on SPIO NWs, modification of hydroxyl groups did not diminish C3 opsonization. Moreover, there was no decrease in C3 opsonization after crosslinking and after modification of CL-NW hydroxyls (Figures 5A,B). There is evidence in the literature that the presence of anionic groups on the polysaccharide surface can promote binding of serum factor H, which is a negative regulator of the AP (3, 34). However, modification of hydroxyl groups of CL-NWs with carboxymethyl, acyl, or ethyl sulfonic groups (Figure 5C) did not decrease the level of C3 opsonization in human serum (Figure 5D).

## Discussion

Previous work has confirmed complement activation on a variety of carbohydrate-coated surfaces (29–31) as well as by crosslinked dextran beads [Sephadex (32)]. In this work, we determined the role of surface modification of dextran-coated iron oxides on the efficiency of complement activation in human sera. The fluid phase assays we employed are designed to dissect the role of the AP and calcium-sensitive pathways in the complement activation (7, 10, 40). Using these assays, we found that regardless of the ratio of dextran/Fe used in the preparation of nanoparticles, the complement activation predominantly proceeds *via* the AP, and with some minor contribution of the calcium-sensitive pathways for formulations prepared with a higher ratio of dextran/Fe. LP is likely the predominant calcium-sensitive pathway activated by these formulations due to the presence of polysaccharide coating, but this would need to be determined in a separate study. In addition, we found that particles prepared with low dextran/Fe ratio incite complement *via* only the AP.

Complement activity toward nanosurfaces is generally much higher in human sera than in sera from other species (41). The main reason for such activity is the continuous, but slow activation of the AP due to tick-over, or formation of fluid phase AP convertase C3(H_2_O)Bb, the initial deposition of C3b on the foreign surface (42). Albeit activation of the AP could happen in the fluid phase, a foreign surface provides the scaffold for C3b and properdin binding, which enhances the assembly of the C3bBb convertase and complement amplification (43). Another mechanism of the AP convertase formation could be the direct binding of C3 to the surface (44). Therefore, we reasoned that the modification of nanoparticle hydroxyl groups could block the initial seeding of C3b and hence the AP activation. Previously, Labarre and colleagues showed that the blocking of alcohol groups (main groups that reacts with thioester of C3b) on Sephadex by carboxymethyl residues (32) prevented complement activation. In addition, the same group demonstrated that the presence of sulfate groups on a surface can mitigate complement activation by attracting factor H, the inhibitor of the AP convertase (33). Unlike these findings, we demonstrate that blocking hydroxyl groups for SPIO NWs and crosslinked CL-NWs with alkylating, crosslinking, and negatively charged groups did not decrease C3 opsonization of SPIO NWs in human sera. Based on the inability of dextran hydroxyl substitutions to block C3 opsonization of SPIO NWs and CL-NWs, it is possible that other surface entities could promote the binding of C3, including non-specifically absorbed proteins, and we are currently investigating this possibility. The differences between the above mentioned results and our particles could be related to the differences in the surface nano-architecture, which promote different binding of complement activators and inhibitors, and needs to be investigated further.

In conclusion, our data establish the AP as the critical pathway for many SPIO formulations. In recent years, the AP has shown to be the essential pathway of complement activation in health and disease (45). Due to its key role, the AP represents a unique therapeutic target in many pathological conditions (46–48). For many drug delivery nanoplatforms, the AP has shown to be a critical pathway for complement activation (20, 21, 49), and it is likely that this list will only grow. The future research will focus on specific approaches to block the AP activation, for example, by using properdin-blocking antibodies (50) or natural serum complement inhibitors (51).

## Materials and Methods

### Materials

Iron salts (ferrous and ferric chloride) and 20 kDa dextran (range 15–25 kDa) were from Sigma–Aldrich (St. Louis, MO, USA). Epichlorohydrin, anhydrous DMSO, 2-chloroethanesulfonic acid, chloroacetic acid, and acetic anhydride were from Sigma, 2-(2-methoxyethoxy)acetyl chloride and 2-methoxyethoxymethyl chloride were from Alfa Aesar. Goat anti-human complement C3 polyclonal antibody (catalog No. 0855444) was purchased from MP Biomedicals (Solon, OH, USA). Anti-goat, IRDye 800CW-labeled, secondary antibodies were from LI-COR Biosciences (Lincoln, NE, USA). Copper grids (300 mesh) were purchased from Electron Microscopy Sciences (Hatfield, PA, USA). Sera from normal female subjects were collected by Equitech-Bio (Kerrville, TX, USA) according to the company’s Institutional Review Board Protocol. All blood products and complement proteins were kept aliquoted at −80°C.

### Synthesis and Modification of SPIO Nanoworms

Nanoworms were synthesized using a one-pot Molday and MacKenzie (52) precipitation method as described by us previously (35). The main variation of the protocol was the ratio of dextran and iron salts in the reaction as described in Figure 1. The molar ratio between Fe^2+^ and Fe^3+^ was kept the same. After the synthesis, particles were dialyzed in double distilled water, filtered through a 0.45-μm filter (Millipore), and stored at 4°C. TEM imaging was conducted to visualize the iron oxide core using FEI Tecnai Spirit BioTwin electron microscope (Electron Microscopy Facility at the University of Colorado Boulder). Size and zeta potential measurements of NPs were determined using a Zetasizer Nano ZS (Malvern Instruments Ltd., Malvern, UK). The intensity weighted size distribution peak value was used to report hydrodynamic diameters of NWs.

For dextran shell crosslinking with epichlorohydrin, a two-step procedure was used as described before (35). For modification of dextran hydroxyls, SPIO NWs prepared at low dextran/Fe ratio (3 g dextran per 133.4 mg Fe salts), or the corresponding crosslinked CL-NWs were washed by ultracentrifugation in anhydrous DMSO two times and resuspended in anhydrous DMSO at 5.0 mg/mL (Fe concentration) in a borosilicate glass vial in the presence of 1 mg/mL of 4-dimethylaminopyridine (DMAP). Then, 2 mg/mL of 2-(2-methoxyethoxy)acetyl chloride or 2 mg/mL of 2-methoxyethoxymethyl chloride were added to the nanoparticles under stirring. Nanoparticles were incubated under nitrogen atmosphere with stirring at 37°C overnight, washed 3× in DMSO, 2× in DDW by ultracentrifugation, and resuspended in PBS for complement measurement. For modification with acetic anhydride, chloroacetic acid, or chloroethanesulfonic acid, CL-NWs were resuspended in DDW at 5 mg/mL (Fe concentration), stirred for 30 min in 2N NaOH solution, and then reacted with acetic anhydride (5% v/v), chloroacetic acid (5 mg/mL), or chloroethanesulfonic acid (5 mg/mL) at 37°C overnight with stirring. The particles were washed by ultracentrifugation and resuspended in PBS.

### Complement Activation Studies

Details of human serum preparation, characterization, and functional assessment of complement pathways were described in detail elsewhere (7, 10, 20). Briefly, serum was prepared from freshly collected blood of two healthy volunteers according to procedure by Lachmann (53). C2 was immunochemically depleted from human serum, and the depleted serum was characterized as described elsewhere (7, 10). To measure complement activation *in vitro*, we determined NW-induced rise of serum complement activation products C4d, Bb, C5a, and sC5b-9 using respective Quidel (Quidel, San Diego, CA, USA) ELISA kits according to the manufacturer’s protocols. In all measurements, the volume of NW to normal or C2-depleted sera volume was 1:4. NW-mediated complement activation was further monitored after restoration of C2 (650 μg/mL) in C2-depleted serum. Zymosan (0.2 mg/mL) was used as a positive control for complement activation throughout. Each experiment was repeated three times with sera from two healthy individuals.

### Analysis of Binding of Proteins to Particles

For binding assay of complement C3 and properdin, 1 mg/mL (Fe) SPIO NWs were incubated with fresh serum at 1:3 volume ratio. At the end of incubation, particles were washed three times with 1× PBS by centrifugation at 100,000× *g* at 4°C in 2 mM Ca^2+/^Mg^2+^ supplemented PBS using Beckman Optima TLX ultracentrifuge. The pellets were resuspended in 20 μL PBS, and 2 μL aliquots were applied in triplicate onto a nitrocellulose membrane (Bio-Rad). The membranes were blocked using 5% (w/w) non-fat dry milk in PBS-T (1× PBS with 0.1% v/v Tween^®^ 20) for 1 h at room temperature, probed with corresponding primary antibodies for 1 h at room temperature, followed by washing the membranes three times with PBS-T, and finally 1 h incubation with the corresponding IRDye 800CW-labeled secondary antibodies against the primary antibody species. The signal was visualized using an Odyssey infrared imager (Li-COR Biosciences, Lincoln, NE, USA). The integrated dot intensity in the scanned images was determined from 16-bit grayscale images using ImageJ software and plotted using Prism 6 software (GraphPad Software, Inc., La Jolla, CA, USA). Each experiment was repeated two times using sera from two individuals.

## Ethics Statement

The study used de-identified human sera previously collected by commercial body for research purposes and as such was exempt from institutional review board protocol.

## Author Contributions

GW, FC, and LW performed the experiments; NKB, VMH, and SMM provided reagents; SMM and DS analyzed the data and wrote the manuscript.

## Conflict of Interest Statement

The authors declare that the research was conducted in the absence of any commercial or financial relationships that could be construed as a potential conflict of interest.
